# Autoimmune Skin Diseases in the Era of COVID-19: Pathophysiological Insights and Clinical Implications

**DOI:** 10.3390/microorganisms13092129

**Published:** 2025-09-11

**Authors:** Aikaterini I. Liakou, Eleni Routsi, Kalliopi Plisioti, Eleni Tziona, Dimitra Koumaki, Magdalini Kalamata, Evangelia-Konstantina Bompou, Rozeta Sokou, Petros Ioannou, Stefanos Bonovas, George Samonis, Andreas G. Tsantes, Alexander Stratigos

**Affiliations:** 1 1st Department of Dermatology and Venereology, “Andreas Syggros” Hospital for Skin Diseases, National & Kapodistrian University of Athens Medical School, 16121 Athens, Greece; routsie@gmail.com (E.R.); magda.kalamata@gmail.com (M.K.); valentina.bompou@gmail.com (E.-K.B.); alstrat2@gmail.com (A.S.); 2 Faculty of Medicine and Pharmacy, “Dunarea de Jos” University of Galati, 800008 Galati, Romania; kalliopi.plisioti@gmail.com; 3 Aristotle University School of Medicine, 54124 Thessaloniki, Greece; helentziona@gmail.com; 4 Dermatology Department, University Hospital of Heraklion, 71110 Heraklion, Greece; dkoumaki@yahoo.gr; 5 Neonatal Department, Aretaio Hospital, National and Kapodistrian University of Athens, 11528 Athens, Greece; sokourozeta@yahoo.gr; 6 School of Medicine, University of Crete, 71003 Heraklion, Greecesamonis@med.uoc.gr (G.S.); 7 Department of Biomedical Sciences, Humanitas University, Pieve Emuanuele, 20072 Milan, Italy; sbonovas@gmail.com; 8 IRCCS Humanitas Research Hospital, Rozzano, 20089 Milan, Italy; 9 1st Oncology Department Metropolitan Hospital, Neon Faliron, 18547 Athens, Greece; 10 Laboratory of Hematology and Blood Bank Unit, “Attikon” University Hospital, School of Medicine, National and Kapodistrian University, 12462 Athens, Greece; 11 Microbiology Department, “Saint Savvas” Oncology Hospital, 11522 Athens, Greece

**Keywords:** COVID-19, SARS-CoV-2, autoimmune skin diseases, hidradenitis suppurativa, psoriasis

## Abstract

The COVID-19 pandemic has highlighted intricate associations between SARS-CoV-2 infection and autoimmune skin diseases (ASDs). This review examines the bidirectional relationship between COVID-19 and ASDs including hidradenitis suppurativa, psoriasis, atopic dermatitis, alopecia areata, autoimmune bullous diseases, cutaneous and systemic lupus erythematosus, systemic sclerosis, dermatomyositis, and lichen planus. Current evidence indicates that SARS-CoV-2 may precipitate or worsen ASDs via mechanisms such as molecular mimicry, dysregulated cytokine signaling, and enhanced Th1/Th17 immune responses, leading to loss of self-tolerance and autoantibody production. Epidemiological studies have identified increased incidence and flares of psoriasis, hidradenitis suppurativa, and other ASDs following both COVID-19 infection and vaccination, with mRNA vaccines associated with a higher risk of flare in hidradenitis suppurativa compared with non-mRNA vaccines. Notably, severe COVID-19 is associated with a greater risk of new-onset autoimmune disease, and patients with pre-existing inflammatory skin conditions may have increased susceptibility to SARS-CoV-2 infection but experience less severe COVID-19 courses. These findings underscore the need for ongoing surveillance and mechanistic studies to clarify the immunopathogenic links between SARS-CoV-2 and ASDs and inform management strategies for affected patients in the context of both infection and vaccination.

## 1. Introduction

From the start of the outbreak, several reports appeared on the autoimmune manifestations and autoimmune sequelae of Coronavirus Disease-2019 (COVID-19) infection [1]. The COVID-19 pandemic has brought unprecedented challenges to healthcare systems globally, not only due to the acute effects of SARS-CoV-2 infection, but also because of its broader immunological consequences. Increasing evidence suggests that COVID-19 can act as a trigger or modulator of autoimmune phenomena. Autoimmune skin diseases (ASDs), including psoriasis, hidradenitis suppurativa (HS), atopic dermatitis (AD), and alopecia areata, have shown variable responses during the pandemic, ranging from disease exacerbation to new-onset presentations. Potential mechanisms include direct viral effects, molecular mimicry, dysregulated cytokine responses, and psychological stress. Moreover, the widespread use of immunomodulatory therapies and the introduction of COVID-19 vaccines have raised questions about the safety, efficacy, and impact of these interventions in patients with preexisting or newly emerging ASDs [2]. Despite the growing body of literature, the relationship between COVID-19 and ASDs remains incompletely understood. Current reports are fragmented, and the findings are sometimes conflicting, particularly regarding the bidirectional effects of infection and treatment. A comprehensive synthesis is therefore needed to clarify the clinical patterns, pathophysiological mechanisms, and therapeutic implications. This review aims to summarize the available evidence on the interplay between COVID-19 and autoimmune skin diseases, highlight emerging pathophysiologic insights, and discuss implications for clinical management and vaccination strategies.

## 2. Hidradenitis Suppurativa

The COVID-19 pandemic has significantly impacted the management and progression of chronic inflammatory skin diseases, notably HS. HS is a chronic inflammatory skin disorder characterized by painful nodules, abscesses, and sinus tracts, primarily affecting intertriginous areas. The pathogenesis of HS involves immune dysregulation, with elevated levels of pro-inflammatory cytokines such as tumor necrosis factor-alpha (TNF-α) and interleukin-17 (IL-17) [3,4,5].

The relationship between COVID-19 infection and HS disease activity has been a subject of investigation. Although the exact mechanisms associated between COVID-19 and flares in autoimmune skin diseases remain unclear, viral infections can exacerbate autoimmunity through molecular mimicry, bystander activation, and persistent immune dysregulation. Cytokines such as TNF-α, IL-17, and IL-23 drive inflammation in HS patients, with COVID-19 infection potentially enhancing this response through increased IL-1β production, IL-17 activation, and inflammasome activation [6,7,8]. COVID-19-induced cytokine release syndrome, marked by excessive interleukin and TNF-α secretion, may disrupt immune homeostasis, triggering disease exacerbation [9,10,11,12]. Moreover, limited access to healthcare services during lockdowns led to delays in diagnosis and treatment, adversely affecting disease control. Additionally, lifestyle changes such as reduced physical activity and dietary alterations contributed to weight gain, potentially exacerbating HS severity. A study highlighted that decreased physical activity during lockdowns increased the obesity rates, a known exacerbating factor for HS [13]. Another study reported that while most HS patients infected with COVID-19 experienced no change in disease activity, approximately 18.8% noted a marked increase in pain and the development of new inflammatory lesions during infection. These findings suggest that COVID-19 may exacerbate HS in a subset of patients, potentially due to the systemic inflammatory response triggered by the virus [14].

The management of biologic treatments in patients with COVID-19 has been debated due to concerns about the potential negative effects on infection progression. COVID-19 follows a biphasic course, beginning with an antiviral phase and transitioning to a hyperinflammatory state. Cytokines like TNF-α, IL-1, and IL-6 dominate the inflammatory phase, while IL-15, interferons, and other cytokines are crucial for viral clearance. These pathways suggest that anti-TNF-α and anti-IL-17 therapies may not directly impair antiviral responses. Clinical guidance on the continuation or discontinuation of biologics should be individualized and is supported by phase 3 trials showing no increased infection risk in HS patients treated with adalimumab [15,16,17].

Regarding COVID-19 susceptibility, the current evidence does not indicate that HS inherently increases the risk of contracting SARS-CoV-2. However, concerns have been raised regarding whether HS itself predisposes patients to more severe COVID-19 outcomes. A retrospective study compared HS patients who contracted COVID-19 with non-HS COVID-19 patients [18]. The study found that HS patients who contracted COVID-19 were significantly more likely to experience complications (35% vs. 7%; *p* = 0.001) and require COVID-19 treatment (37% vs. 7%; *p* = 0.0001) compared with non-HS COVID-19 patients. However, it is important to note that HS patients often have comorbidities such as obesity and cardiovascular disease, which are established risk factors for severe COVID-19. Therefore, the presence of these comorbidities, rather than HS itself, may contribute to increased risk.

Although the introduction of COVID-19 vaccines has been pivotal in controlling the pandemic, there have been reports of HS exacerbations following vaccination. Recent studies have investigated the impact of COVID-19 vaccination on HS, raising questions about safety and potential disease exacerbation. In a retrospective study of 250 vaccinated HS patients, mRNA vaccines were associated with a higher risk of post-vaccination flares compared with non-mRNA vaccines [19,20]. However, as this was a single-center study with a limited sample size, the findings should be interpreted with caution and require confirmation in larger cohorts. Notably, HS patients on biologic therapy were less likely to develop flares post-vaccination, suggesting a protective role of biologics. A small case series, including reports by Martora et al. and Alexander et al., described HS exacerbations post-vaccination. However, the authors of both studies concluded that these flares did not justify contraindicating further doses [21,22]. Larger studies, such as a multicenter retrospective cohort by Pakhchanian et al., found no significant increase in vaccine-related adverse events among HS patients, even those on systemic therapy. Clinicians should monitor patients for potential flares and optimize disease management pre- and post-vaccination. Further epidemiological and mechanistic studies are needed to clarify the relationship between COVID-19 vaccination and HS [23].

While data specific to HS are limited, extrapolation from other inflammatory conditions suggests that patients on biologic therapies can mount adequate immune responses to vaccines. Consultation with healthcare providers is essential to determine the optimal timing of vaccination, especially concerning ongoing immunosuppressive treatments. Ongoing research and patient registries, such as the Global HS COVID-19 Registry, are essential in deepening our understanding and guiding management strategies for HS patients during this unprecedented time [24,25,26].

## 3. Psoriasis

Psoriasis is a chronic autoimmune skin disorder characterized by the hyperproliferation of keratinocytes and systemic inflammation. The COVID-19 pandemic has introduced complexities in managing psoriatic patients, particularly concerning the interplay between the disease, its treatments, and SARS-CoV-2 infection [27,28,29].

Emerging evidence suggests that COVID-19 infection may act as a triggering factor for psoriasis exacerbations, likely through the dysregulation of immune pathways implicated in the disease. The relationship between psoriasis and COVID-19 is multifaceted. Ιn psoriasis, the Th17-driven inflammatory response, characterized by elevated levels of IL-23, IL-17, and TNF-α, is amplified during COVID-19 infection, triggering disease flares [30,31,32]. Psoriasis flares in the context of COVID-19 infection are primarily attributed to the virus-induced hyperinflammatory state, commonly referred to as a “cytokine storm”. COVID-19 infection leads to the excessive production of pro-inflammatory cytokines, including ILs and TNF-α, which are pivotal in the pathogenesis of psoriasis. This overproduction can disrupt the delicate immune balance, potentially triggering or exacerbating psoriatic lesions. Additionally, the binding of the SARS-CoV-2 spike protein to the ACE2 receptor results in its downregulation, leading to increased levels of angiotensin II. Elevated angiotensin II can further promote inflammation, contributing to psoriatic flare-ups. Moreover, the systemic inflammation associated with COVID-19 may amplify the availability of self-antigens through mechanisms like antigen mimicry and epitope spreading, further fueling psoriatic disease activity. Moreover, the psychological impact of the pandemic on psoriatic patients has also been significant. Stress is a well-known trigger for psoriasis exacerbations, and the global crisis has undoubtedly heightened the stress levels among individuals [29,33,34]. Several case reports have documented post-COVID-19 flares of psoriasis including guttate, pustular, and severe plaque variants [35,36,37,38,39]. Although current data are limited to individual case observations, these findings underscore the need for heightened clinical awareness and further investigation into the immunological interplay between COVID-19 and chronic inflammatory dermatoses such as psoriasis [39].

The management of psoriasis during the pandemic has seen varied approaches. Notably, studies reported that teledermatology services effectively managed psoriasis patients, reducing the need for face-to-face consultations and thereby minimizing potential exposure to the virus. This approach not only ensured continuity of care, but also maintained high patient satisfaction and compliance. Teledermatology emerged as an important tool for psoriasis care, ensuring the continuity of treatment and minimizing exposure risks. Studies reported high patient satisfaction, improved treatment adherence, and reduced need for in-person visits [40,41,42,43]. However, limitations included uneven access to technology and challenges in assessing subtle clinical changes remotely. In many cases, the COVID-19 pandemic led to treatment disruptions due to concerns about immunosuppression, prompting some individuals to discontinue or delay biologic therapies, potentially leading to disease flares. While initial fears prompted treatment interruptions, accumulating evidence suggests that biologics targeting IL-17 and IL-23 do not increase COVID-19 severity. Observational studies and registry data have reported no higher rates of infection or adverse outcomes among psoriasis patients receiving biologics compared with those not on systemic therapy [40]. Nonetheless, the evidence is largely derived from observational cohorts, and randomized trial data remain lacking. Therefore, many patients and clinicians opted to continue biologic therapies, given the lack of definitive evidence linking these treatments to increased COVID-19 severity. Based on the current literature, the continuation of biological therapy under medical supervision is advised, balancing the benefits of disease control against potential infection risks [40].

Early in the pandemic, concerns arose regarding the susceptibility of psoriatic patients to COVID-19, especially those on immunomodulatory therapies. However, accumulating evidence suggests that psoriasis itself does not inherently increase the risk of contracting COVID-19. Instead, comorbidities frequently associated with psoriasis, such as obesity, diabetes, and cardiovascular diseases, have been identified as significant risk factors for severe COVID-19 outcomes [27,28]. A study indicated that among psoriasis patients infected with SARS-CoV-2, 5.8% required hospitalization, and 23.6% experienced psoriasis exacerbation due to the infection [29].

Vaccination against COVID-19 has been a pivotal strategy in controlling the pandemic. For psoriatic patients, concerns regarding vaccine efficacy and potential disease flares post-vaccination have been prevalent. Current evidence indicates that mRNA COVID-19 vaccines are safe for individuals with psoriasis including those on immunomodulatory therapies [44].

The psychological impact of the pandemic on psoriatic patients has also been significant. Stress is a well-known trigger for psoriasis exacerbations, and the global crisis has undoubtedly heightened stress levels among individuals. A study conducted in Denmark highlighted that individual with psoriasis experienced worsened mental well-being during the pandemic compared with healthy individuals, underscoring the need for holistic patient care that addresses both physical and psychological health [45]. 

## 4. Atopic Dermatitis

The relationship between COVID-19 infection and AD remains complex. AD is a chronic inflammatory condition resulting from a combination of skin barrier dysfunction and immune dysregulation. Two main theories explain its pathogenesis: the “outside-in” hypothesis, which suggests that environmental allergens initiate the disease by weakening the skin barrier and promoting IgE sensitization, and the “inside-out” hypothesis, which proposes that genetic factors—most notably mutations in the filaggrin (FLG) gene—lead to impaired barrier function and subsequent immune activation. During the COVID-19 pandemic, AD flares have been reported more frequently, likely due to enhanced type 2 cytokine activity (IL-4, IL-13, IL-31), increased psychological stress, and repetitive hand hygiene practices that further compromise the skin. Exacerbations may be managed with topical corticosteroids, while systemic treatments such as JAK inhibitors are considered for more severe or persistent disease [46,47]. Skin biopsies from AD patients also show a predominance of Th2-derived cytokines including IL-4 and IL-13, implicated in pathogenesis [48]. The Th2/Th1 imbalance observed in AD, which promotes IgE-mediated hypersensitivity, may be disrupted by COVID-19 infection, leading to AD exacerbations [49,50]. In an investigation conducted in the Netherlands, 26% of the participants with AD reported a deterioration in symptoms during active COVID-19 infection [51]. Another study revealed that 43% of AD patients experienced disease flare-ups post-COVID-19, with many requiring treatment adjustments. Interestingly, exacerbation in patients receiving immunosuppressives was less pronounced, possibly due to the protective benefits of these drugs against virus-induced inflammatory cascades [47,52,53,54,55]. Epidemiological evidence further supports a potential link between COVID-19 and new-onset AD. A retrospective study reported that individuals with a history of SARS-CoV-2 infection had a 33% higher likelihood of developing AD compared with matched controls [54]. However, this study relied on registry data and lacked long-term follow-up, so the findings should be interpreted with caution.

In conclusion, COVID-19 may exacerbate AD through several interrelated mechanisms, including heightened type 2 cytokine activity (IL-4, IL-13, IL-31), skin barrier impairment from repetitive hand hygiene practices, and increased psychological stress, all of which converge to disrupt immune homeostasis and promote disease flares.

## 5. Alopecia Areata

Recent research has explored the link between COVID-19 and alopecia areata (AA), revealing that the virus may act as a catalyst for the onset or exacerbation of the condition. The exact mechanisms linking COVID-19 to AA remain under investigation. The current mechanism of AA is not clear. From a biological perspective, SARS-CoV-2 infection may exacerbate or trigger AA through IFN-mediated antiviral responses that disrupt hair follicle immune privilege, upregulate MHC class I expression, and activate pro-inflammatory cytokines such as IL-6 [56]. In parallel, psychological stress, which was pervasive during the pandemic, may act as an additional aggravating factor. Stress is known to influence immune responses and exacerbate autoimmune conditions and therefore may contribute independently or synergistically to AA flares during the COVID-19 era [57,58]. The systemic inflammatory response caused by the virus could also provide a potential and plausible explanation [59].

COVID-19 has been shown to affect AA recurrence, as higher relapse rates (44%) were noted among those who contracted SARS-CoV-2 compared with the control group (12%) [60]. Other data support that individuals developed AA 1 to 2 months after infection from COVID-19, suggesting an association between infection and disease emergence [61]. Epidemiological studies also support this association. For instance, large-scale research in South Korea found an increased incidence of AA among individuals who had recovered from COVID-19 [58]. Another large retrospective cohort highlighted that this correlation of disease incidence was also tightly interconnected to the severity of COVID-19 [62,63]. While these findings are compelling, the relationship between COVID-19 and AA remains complex, and for the most part unknown. It is important to note that the majority of available evidence consists of case reports and retrospective cohorts, which may be subject to publication bias. While emerging data suggest a possible link between COVID-19 and AA, this relationship remains complex and is not yet fully elucidated. Larger, prospective studies are needed to disentangle the biological effects of SARS-CoV-2 infection from psychological stressors and to minimize the influence of publication bias inherent in early reports. Unraveling this connection in the post-COVID-19 era, when our information and understanding of both entities increases, could significantly improve the management of AA in patients affected by COVID-19. The delineation of the relationship between COVID-19 and AA could provide insights into how viral infections impact autoimmune diseases.

## 6. Autoimmune Bullous Diseases

Autoimmune bullous diseases (AIBDs), such as pemphigus vulgaris and bullous pemphigoid, arise when autoantibodies target structural proteins in the skin, leading to blister formation. Emerging data suggest that SARS-CoV-2 infection can trigger or worsen AIBDs through immune dysregulation: viral-induced cytokine release and molecular mimicry between viral peptides and skin antigens—as evidenced by shared epitope sequences with bullous pemphigoid—may provoke autoantibody production [64]. Several case reports have reported that in patients with pre-existing pemphigus or bullous pemphigoid, the onset of COVID-19 has been associated with flare-ups or new cases [61,65]. The immune system’s heightened inflammatory response during COVID-19 infection may contribute to the worsening of these conditions, as it can potentially activate autoimmune mechanisms, leading to the breakdown of skin structures that result in antigen shedding, immune activation, and ultimately blister formation and manifestation of the clinical entities of autoimmune bullous disease of the skin [66]. In addition to the above, the use of corticosteroids and other immunosuppressive medications (e.g., CD20-directed agents) commonly prescribed to manage severe pemphigus, and bullous pemphigoid may further complicate the management of COVID-19 infection [67]. B-cell depletion and subsequent humoral immunity impairment make patients highly susceptible to a broad range of infections, including COVID-19, and possibly influence the severity of the disease course [68]. Current expert guidance suggests that abrupt discontinuation of immunosuppressive therapy in patients with AIBDs should generally be avoided, as uncontrolled disease activity may itself increase morbidity. Instead, therapy should be tailored individually: patients on systemic corticosteroids may require careful dose adjustments, while those on B-cell–depleting agents such as rituximab should be closely monitored, with the consideration of delaying treatment in stable disease during active SARS-CoV-2 infection [68]. This has been documented in clinical evidence supporting that patients on long-term immunosuppressive therapy for autoimmune blistering diseases may experience more severe COVID-19 infection [69]. It should be noted that the majority of current evidence derives from case reports and small retrospective studies, which are inherently subject to publication bias and limited generalizability. As a result, the association between COVID-19 and AIBDs should be interpreted with caution until supported by larger prospective studies.

## 7. Cutaneous and Systemic Lupus Erythematosus

Cutaneous lupus erythematosus (CLE) encompasses autoimmune skin disorders that can be influenced by various environmental factors including viral infections. Emerging evidence suggests a correlation between SARS-CoV-2 infection and the onset or exacerbation of CLE [70]. Although SARS-CoV-2 has been associated with the induction of autoantibodies such as ANA, lupus anticoagulant, and anti-Ro/SSA in some reports, these findings remain preliminary and do not establish a direct pathogenic link to lupus or systemic sclerosis. The proposed mechanisms should therefore be interpreted as speculative hypotheses rather than confirmed pathways [71,72].

A few case reports have described an association between COVID-19 and CLE. A 67-year-old woman with pre-existing CLE developed a Rowell syndrome-like flare approximately two weeks after contracting SARS-CoV-2 [73]. Another case report presented a patient with a rare variant of CLE, namely lupus panniculitis, as the primary manifestation of SLE stimulated by COVID-19 infection [74]. Although the clinical relevance of this evidence remains limited, the association between LE and COVID-19 is expected to be further delineated as our knowledge and experience with the virus expands. Overall, while case reports suggest a possible association between COVID-19 and lupus manifestations, the evidence remains anecdotal, and mechanistic explanations are speculative. More robust studies are needed to determine whether SARS-CoV-2 acts as a true disease trigger or whether the observed associations reflect coincidental findings.

## 8. Systemic Sclerosis

There have been reports of new-onset SSc following COVID-19 infection. SS, defined by progressive fibrosis and vascular dysfunction, parallels COVID-19-related endothelial injury and hyperinflammation [75,76]. Severe acute respiratory syndrome coronavirus 2 (SARS-CoV-2) infection could thus accelerate fibrotic processes, contributing to worsened outcomes in SSc patients [77]. Interestingly, a case report described a female patient who developed systemic sclerosis after COVID-19 infection, highlighting the potential for SARS-CoV-2 to trigger autoimmune responses leading to SSc development. Additionally, the SARS-CoV-2 spike protein has been found to accelerate systemic sclerosis by promoting fibrosis through the upregulation of pathways involved in inflammatory responses and autoantibody formation and secretion [78]. A major SSc complication is interstitial lung disease (ILD). Therefore, ILD could potentially predispose SSc patients to severe COVID-19 infection associated with poor outcomes [78]. Additionally, considering the concomitant use of immunosuppressive therapies in severe SSc, the risk could increase even more [75]. COVID-19 can cause severe pneumonia with radiological features of interstitial fibrosis, similar to ILD secondary to SSc, thereby complicating the clinical scenario by presenting a unique challenge in differential diagnosis between the two [79].

## 9. Dermatomyositis

Dermatomyositis (DM) is a systemic autoimmune disease characterized by skin manifestations and proximal muscle weakness, with COVID-19 increasingly reported as a potential trigger for both new-onset disease and flares of pre-existing DM. Evidence remains limited and largely anecdotal. For example, single case reports describe new-onset DM following SARS-CoV-2 infection and flare-ups in young patients after even mild COVID-19 illness [80,81]. While these reports suggest a possible association, the reliance on case-based data limits generalizability and may be influenced by publication bias.

Mechanistically, autoantibodies play a central role. Notably, *anti-melanoma differentiation-associated gene 5* (MDA5) antibodies, which are linked to severe forms of DM with interstitial lung disease, have also been associated with worse COVID-19 outcomes and increased mortality [82]. The management of DM during the COVID-19 pandemic requires balancing immunosuppression with infection risk. Data suggest that maintaining disease control is critical, as uncontrolled DM poses greater risks than potential viral complications. Corticosteroids should be used at the lowest effective dose, and steroid-sparing immunosuppressive agents (e.g., methotrexate, azathioprine, mycophenolate mofetil) may be considered [83]. B-cell depleting therapies and high-dose corticosteroids should be used with caution, particularly in patients at high risk for severe COVID-19. Vaccination and preventive measures remain essential adjuncts to care [83]. Further research is needed to provide more insights into the optimal management strategies for DM patients amidst the pandemic.

## 10. Lichen Planus

Lichen planus (LP) is a group of chronic inflammatory diseases that mainly affect middle-aged adults. While LP primarily includes skin and oral mucosa, other mucous membranes and skin appendages, such as nails and hair, can also be damaged [83,84,85,86,87]. LP is increasingly considered a T cell-mediated autoimmune condition in which cytotoxic CD8+ T cells induce basal keratinocyte apoptosis, producing the characteristic interface dermatitis [88,89,90]. Although the exact etiology remains unclear, both infectious triggers (e.g., herpes simplex, varicella zoster, HPV-16) and immune dysregulation have been implicated [90,91,92]. However, the precise relationship between viruses and LP remains an area of ongoing research.

In the context of COVID-19, several overlapping immunological pathways may contribute to LP pathogenesis. SARS-CoV-2 infection stimulates robust Th1 and Th17 immune responses and cytotoxic CD8+ T-cell activity, all of which can promote keratinocyte apoptosis, a hallmark of LP [93,94,95,96,97,98]. Molecular mimicry between SARS-CoV-2 antigens and epidermal self-antigens may further drive autoimmune cytotoxicity [92,99,100]. Additionally, ACE2 receptors expressed in keratinocytes and oral mucosal cells provide a potential portal for viral interaction, amplifying local inflammation [101,102,103]. Together, these mechanisms suggest that SARS-CoV-2 infection could act as a trigger for LP in genetically or immunologically predisposed individuals, though definitive causal links remain unproven. Another hypothesis is that molecular mimicry is responsible for triggering the autoimmune cytotoxic T cell (CTL) and Th1 responses that mediate LP in COVID-19 infection [92,100]. The SARS-CoV-2 antigen has demonstrated cross-reactivity with multiple endogenous human antigens including those found on the basal keratinocytes of the epidermis. Another important consideration is that the role of ACE2 receptors for host cell entry may be implicated, as these receptors are abundant in cells of the skin and oral mucosa. The binding of the SARS-CoV-2 spike protein to ACE2 receptors on epidermal cells may trigger Th1 recruitment and the subsequent autoimmune cascade responsible for LP pathogenesis in infection with SARS-CoV-2 [101,102,103].

Clinically, sporadic reports describe both new-onset and exacerbations of LP after COVID-19 infection [96]. Interestingly, more cases have been documented following COVID-19 vaccination, though these remain rare and are primarily limited to case reports and small series [97]. Given the scale of the global vaccination efforts, the absolute risk appears extremely low, and causality has not been established. Current evidence therefore does not alter existing vaccination recommendations, which remain strongly favorable for patients with chronic inflammatory diseases.

**Figure 1:**
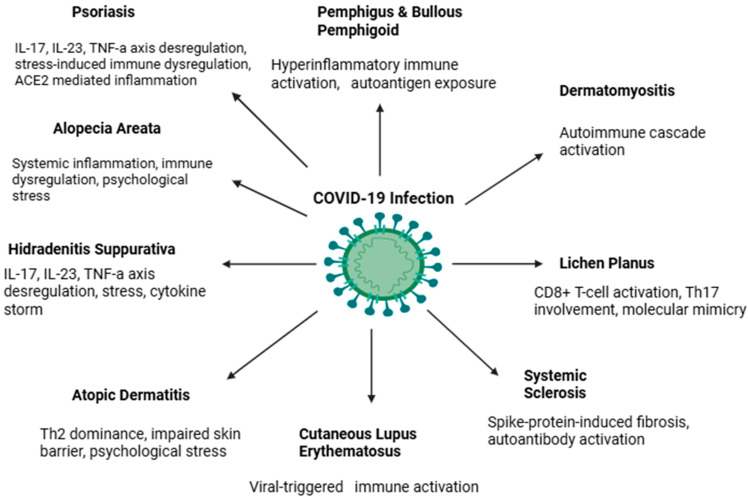
Potential mechanisms linking COVID-19 infection with the development or exacerbation of dermatologic autoimmune and inflammatory skin diseases.

Finally, there are concerns that immunocompromising comorbidities, including hypertension, diabetes and vitamin D deficiency, are risk factors that may increase susceptibility to LP after COVID-19 infection. Diabetes and hypertension have been identified as risk factors for oral LP development and COVID-19 mortality, and vitamin D has been found to modulate Th1 cells and regulate *T*-cell-mediated immune activity [99]. However, further systematic studies are required to disentangle these associations from coincidental findings. The findings of autoimmune and chronic inflammatory skin diseases affected by COVID-19 infection are summarized in Table 1 and Figure 1.

## 11. COVID-19 and Vaccines

Emerging reports indicate cutaneous adverse events associated with COVID-19 vaccination including new-onset conditions and exacerbations of pre-existing dermatoses [104]. Both mRNA and adenoviral vector SARS-CoV-2 vaccines activate innate immune sensors through intrinsic adjuvant activity, leading to the production of IFNs and pro-inflammatory cytokines [105]. Adenoviral vectors activate Toll-like receptors (TLRs) 2 and 9, inducing cytokine expression through macrophage activation in the liver and spleen, while also eliciting robust CD4+ Th1 and humoral immune responses. In contrast, mRNA vaccines activate inflammation through TLRs 3, 7, and 8, with lipid nanoparticles (LNPs) exhibiting immunostimulatory properties even in the absence of mRNA cargo [106]. Essentially, both vaccine platforms predominantly induce a Th1-skewed immune response, characterized by the increased production of TNF-α, IFN-γ, and IL-2. In this context, this Th1-dominant environment necessitates the awareness of potential flares in immune-mediated skin conditions and connective tissue diseases, where Th1-mediated mechanisms are implicated in pathogenesis [107].

Additionally, the incidence of de novo or relapsing autoimmune skin diseases appears higher following mRNA-based vaccines, potentially due to their elevated immunogenicity compared with viral vector and inactivated vaccines [108]. In patients with hidradenitis suppurativa, a recent study has shown that those who received mRNA vaccines were 3.5 times as likely to develop flares following vaccination compared with patients who received non-mRNA vaccines, indicating that mRNA COVID-19 vaccines could be associated with hidradenitis suppurativa flares [109]. Moreover, a study by Jęśkowi-ak-Kossakowska et al. suggests that adenoviral vector vaccines may present fewer adverse effects, possibly due to the organism’s faster adaptation to this technology [110]. On the other hand, inactivated viral vaccines, while weaker inducers of CD8+ T cell responses compared with mRNA and adenoviral vector vaccines, may therefore present a lower theoretical risk of triggering Th1-driven autoimmunity. Indeed, observational data suggest that inactivated vaccines are generally well-tolerated in patients with autoimmune and chronic inflammatory conditions. However, their immunogenicity is influenced by the choice of adjuvant, and large-scale comparative data in dermatologic autoimmune diseases remain scarce. This underscores the need for future studies to clarify their precise safety and efficacy profile in this population [111].

Finally, a study by Sprow et al. found that most patients with autoimmune skin diseases effectively tolerated the COVID-19 vaccine. Although 14.8% of fully vaccinated participants reported exacerbations of autoimmune symptoms after vaccination, only 6.7% required an escalation in treatment, indicating that the overall safety and tolerability of the COVID-19 vaccine in this population remain high, despite the presence of underlying autoimmune conditions [108]. Importantly, despite reports of cutaneous flares, the overall safety and tolerability of COVID-19 vaccines in patients with autoimmune skin diseases remain high, as the vast majority of individuals tolerated vaccination without clinically significant worsening of disease activity. These findings are summarized in Table 2.

## 12. Conclusions

Cutaneous manifestations of COVID-19 represent a heterogeneous spectrum, and their prognostic significance depends on the specific type of lesion rather than their mere presence. Early recognition of characteristic skin findings can enable dermatologists to contribute to timely COVID-19 diagnosis, particularly in patients with mild or asymptomatic infection. At present, the impact of pre-existing autoimmune skin diseases on COVID-19 severity and mortality remains uncertain, underscoring the importance of long-term monitoring to detect potential disease exacerbations or the development of new autoimmune connective tissue disorders. Vaccination with non-living platforms, including mRNA vaccines, is generally safe and effective in reducing severe outcomes, though a minority of patients may experience disease flares or new-onset autoimmune conditions. This highlights the need for individualized vaccination planning and close follow-up in patients with autoimmune skin diseases. Importantly, the lessons learned from the impact of COVID-19 on autoimmune skin disorders can serve as a framework for anticipating and mitigating similar complications in future pandemics. Strengthening surveillance systems, fostering multidisciplinary collaboration, and ensuring early dermatologic recognition may reduce morbidity and improve outcomes in comparable global health crises.

## Figures and Tables

**Table 1:** Autoimmune and chronic inflammatory skin diseases affected by COVID-19 infection.

Disease	Impact of COVID-19 Infection	Proposed Mechanisms	Notes
Psoriasis	New onset and exacerbation reported	IL-17, IL-23, TNF-a axis dysregulation; stress-induced immune dysregulation, ACE2 mediated inflammation	Exacerbations more common with prior history; may be influenced by treatments
Hidradenitis Suppurativa	Exacerbation common	IL-17, IL-23, TNF-a axis dysregulation, stress, cytokine storm	Disease flares may be related to COVID-induced cytokine storm
Atopic Dermatitis	New onset and flare-ups	Th2 dominance; impaired skin barrier, psychological stress	Severe flares reported in previously well-controlled cases
Alopecia Areata	New onset or exacerbation	Systemic inflammation, immune dysregulation, psychological stress	Onset often 1–2 months post-infection; relapse rate higher than controls
Pemphigus and Bullous Pemphigoid	Flare-ups or new onset	Hyperinflammatory immune activation, autoantigen exposure	Immunosuppressants may increase infection risk/severity
Cutaneous Lupus Erythematosus	Exacerbation or rare new onset	Viral-triggered immune activation	Limited to case reports
Systemic Sclerosis	New onset; possible ILD worsening	Spike-protein-induced fibrosis; autoantibody activation	ILD may mask or worsen COVID-related respiratory symptoms
Dermatomyositis	Disease onset and exacerbation	Autoimmune cascade activation	Flare-ups also after mild cases
Lichen Planus	Sporadic new onset	CD8+ T-cell activation, Th17 involvement, molecular mimicry	Vitamin D deficiency, HTN, diabetes may be co-factors

Abbreviations: HTN, hypertension, ILD, interstitial lung disease, IL, interleukin, TNF, tumor necrosis factor, ACE, angiotensin-converting enzyme.

**Table 2:** Autoimmune skin diseases and COVID-19 vaccination.

Vaccine Platform	Mechanism of Immune Activation	Dominant Cytokines/Immune Responses	Cutaneous Adverse Events	Autoimmune Flare Risk
**mRNA Vaccines**	Activation of TLRs 3, 7, 8 via mRNA Intrinsic adjuvanticity of lipid nanoparticles Type I IFN production	Th1 polarization: ↑ TNF-α, ↑ IFN-γ, ↑ IL-2 Robust CD8+ T-cell activation	Higher prevalence of psoriasis, lichen planus, lupus flares Localized erythema	Elevated due to Th1 skewing Strong immunogenicity linked to higher autoimmune risk
**Adenoviral Vector Vaccines**	TLRs 2, 9 activation Macrophage activation in liver/spleen Stimulation of CD4+ Th1 and antibody production	Th1-dominant: ↑ TNF-α, ↑ IFN-γ, ↑ IL-2 Moderate CD8+ T-cell activation	Lower CAE incidence Dermatitis, mild urticaria	Reduced due to faster immune adaptation Milder flares compared to mRNA vaccines
**Inactivated Virus Vaccines**	Weak innate activation via adjuvants Dependent on antigen formulation	Limited Th1 polarization Weak CD8+ T-cell activation	Minimal CAEs Mild transient rashes	Lower risk due to weaker immune activation Rare autoimmune flare cases

Abbreviations: ASD, autoimmune skin diseases, TNF-α, tumor necrosis factor-a, IFN, interferon, TLR, Toll-like receptors, CAE, cutaneous adverse events. ↑, increase

## Data Availability

No new data were created or analyzed in this study. Data sharing is not applicable to this article.
